# Direct Detection of Toxic Contaminants in Minimally Processed Food Products Using Dendritic Surface-Enhanced Raman Scattering Substrates

**DOI:** 10.3390/s18082726

**Published:** 2018-08-19

**Authors:** Hannah Dies, Maria Siampani, Carlos Escobedo, Aristides Docoslis

**Affiliations:** 1 Department of Chemical Engineering, Queen’s University, Kingston, ON K7L 3N6, Canada; 9hed@queensu.ca (H.D.); ce32@queensu.ca (C.E.); 2 Department of Chemistry, University of Ioannina, GR-54110 Ioannina, Greece; mariasiampani16@gmail.com

**Keywords:** surface-enhanced Raman scattering, nanoparticles, thiram, melamine, pesticides, food quality monitoring

## Abstract

We present a method for the surface-enhanced Raman scattering (SERS)-based detection of toxic contaminants in minimally processed liquid food products, through the use of a dendritic silver nanostructure, produced through electrokinetic assembly of nanoparticles from solution. The dendritic nanostructure is produced on the surface of a microelectrode chip, connected to an AC field with an imposed DC bias. We apply this chip for the detection of thiram, a toxic fruit pesticide, in apple juice, to a limit of detection of 115 ppb, with no sample preprocessing. We also apply the chip for the detection of melamine, a toxic contaminant/food additive, to a limit of detection of 1.5 ppm in milk and 105 ppb in infant formula. All the reported limits of detection are below the recommended safe limits in food products, rendering this technique useful as a screening method to identify liquid food with hazardous amounts of toxic contaminants.

## 1. Introduction

The use of chemicals in food preparation has increased tremendously with industrial growth, as has knowledge of the health hazards of these chemical additives, resulting in a need to be able to reliably detect them in downstream consumable products [1]. Food safety analysis is a challenging task, requiring the detection of small molecules frequently at ultralow concentrations, in complex mixtures, and occasionally in environments that may be considered harsh for sensitive laboratory equipment. Ideally, it must also be rapid, cost-effective, and user-friendly, such that minimally trained users in a variety of environments can test for toxic materials in food products [2]. Currently, most methods of food safety analysis require the use of high performance liquid chromatography (HPLC) for separation combined with ultraviolet or mass spectrometry for detection—methods that although robust and reliable, are time-consuming, expensive, and require a dedicated lab with trained personnel [3,4]. 

Surface-enhanced Raman scattering (SERS) has emerged in recent years as a promising method for non-destructive, rapid, chemically-specific, and ultrasensitive small molecule detection [4]. Compared with HPLC-MS, it offers the important advantage of portability—SERS can readily be made compatible with portable laser detection systems, and does not necessitate the use of harsh solvents or high-power sources. SERS can achieve signal enhancement up to 10^14^ times over spontaneous Raman spectroscopy through two mechanisms: (1) a dominant electromagnetic mechanism, whereby surface plasmon resonances on nanostructured metallic surfaces locally enhance the electromagnetic field, and (2) a lesser charge transfer/chemical enhancement, involving direct bonding between the analyte and the SERS surface [5]. SERS has been experimentally demonstrated to be chemically specific and sensitive enough for single molecule detection [6,7,8]; however, the practical application of SERS faces some technical challenges, predominantly associated with the fabrication of reproducible, reliable, and robust SERS-active surfaces. SERS is typically realized through either: (1) a solution-based method, whereby colloidal nanoparticles associate with the analyte enabling its detection, or (2) a surface-based method, whereby a metallic nanostructured surface is prefabricated, and an analyte solution is either dropcast or adsorbed onto the surface for sensing. The former method, while convenient, can suffer from a low density/inhomogeneity of SERS-active structures in solution, due to uncontrolled aggregation of the nanoparticles [9,10]. The latter method can further be divided into top-down or bottom-up methods for fabrication of SERS-active nanostructures [9]. Top-down techniques involve patterning or etching of metals using techniques including electron beam lithography, focused ion beam milling [11], or metal film over nanosphere patterning [12], while bottom-up techniques involve the use of nanoparticle or ion-based building blocks to organize SERS-active nanostructures [9]. 

Recently, we have presented the results of a newly developed method for preparing highly sensitive SERS-active surfaces, produced through an electric field-guided assembly of nanoparticles from a colloidal suspension [13]. This method requires only a low power AC voltage source, a planar microelectrode array, a small droplet of nanoparticle suspension, and can be completed in approximately 10 min. Here, we present a systematic experimental study that thoroughly details a protocol for how these sensing devices may be employed in the detection of toxic contaminants in complex food matrices. In addition, to overcome some of the challenges inherent in sensing chemicals in complex food matrices, we demonstrate: (1) the use of an ionic monolayer for the stabilization of our nanoparticulate SERS substrate against mixtures containing high amounts of surface active agents (e.g., milk proteins); and (2) a method for the simple and quick (<45 min) processing of milk to remove the fat and precipitate the proteins for SERS analysis. In apple juice, we achieve the detection of a fruit pesticide, thiram, with no sample processing or surface modification.

## 2. Materials and Methods

### 2.1. Materials

Silver nanoparticles of 50 nm in diameter, stabilized in 2 mM citrate were obtained from Cytodiagnostics Inc., Burlington, ON, Canada. Nanoparticles were concentrated by 15 times prior to their use via centrifugation at 1800 g for 30 min, followed by removal of the supernatant to reach a final concentration of 2.9 × 10^11^ particles/mL. Melamine (99%), thiram (Pestanal^®^, analytical standard), rhodamine 6G, and poly-L-lysine solution (0.1% *w*/*v* in H_2_O) were obtained from Sigma-Aldrich, Oakville, ON, Canada. Acetonitrile (99.9%) was obtained from Fisher Scientific, Ottawa, ON, Canada. Polished silicon wafers of 4” in diameter, with a thermally grown 0.5 μm thick SiO_2_ layer were acquired from University Wafer (South Boston, MA, USA). Millipore^®^ water (18.2 MΩ·cm) was used throughout the experiments. Milk (1%), infant formula, and apple juice were purchased from a local grocery store. 

### 2.2. SERS Substrate Fabrication and Characterization

The fabrication of microelectrodes was performed at Nanofabrication Kingston (NFK, Innovation Park, Kingston, ON, Canada). Microelectrode patterning was performed via maskless photolithography (IMP maskless photolithography) on silicon wafers, and microelectrode deposition was performed by electron beam metal film evaporation and liftoff. SU-8 (MicroChem Corp., Westborough, MA, USA), a negative photoresist, was used for photolithography. A 5 nm layer of chrome was used to enable the adhesion of the deposited 100 nm Au layer to the silicon substrate. 

For SERS substrate preparation, a 10 μL sample of concentrated nanoparticle solution was deposited with a micropipette on the center of the microelectrodes. The collection was run for 12 min at 10 Hz, 2.9 V peak-to-peak, with an imposed 0.5 V DC bias. Following nanoparticle assembly, the chip was rinsed with water and dried with a stream of air. 

Scanning electron microscopy was performed at the Queen’s Facility for Isotope Research, Kingston, ON, Canada, on an MLA 650 FEG environmental SEM, at a voltage of 5.00 kV. Optical microscopy was performed on an Olympus BX-41 microscope. 

### 2.3. Analyte Sample Preparation

Melamine was dissolved in Millipore water at a stock solution of 1000 ppm (7.9 mM) and serially diluted to make solutions of 100 ppm and 10 ppm. These solutions were used to spike milk at a 10:1 (milk to spiking solution) ratio. Thiram was dissolved in acetone at a stock solution of 1000 ppm (4.2 mM) and serially diluted in acetone to make solutions of 100 ppm, 10 ppm, and 1 ppm. These thiram spiking solutions were added directly to apple juice at a ratio of 10:1 (apple juice to spiking solution), and no additional sample preparation was performed. For melamine-spiked milk or infant formula solutions, a simple sample preparation was carried out to precipitate the proteins and skim off the fat: (1) Acetonitrile was added in a 1:1 ratio to the (spiked) milk/infant formula, and the solution was mixed thoroughly with a vortex mixer; (2) the solution was centrifuged for 30 min at 2000 g, and (3) the precipitated protein pellet was discarded. The supernatant was extracted for SERS analysis. 

### 2.4. Surface Modification for Detection in Milk and Infant Formula

For detection in milk and infant formula, the microelectrode surfaces were modified with poly-L-lysine prior to nanoparticle assembly to promote dendrite anchoring to the surface and avoid any remaining unprecipitated protein acting to destabilize the nanostructures. The microchips were first cleaned in a vacuum with an oxygen plasma for 3 min, followed by soaking in 0.01% aqueous poly-L-lysine for 30 min in a humidity-controlled environment. Excess unbound poly-L-lysine was then removed by rinsing with PBS and deionized water. The chip was then dried in a stream of nitrogen. 

### 2.5. Raman Measurements

For the Raman measurements, a HORIBA/Jobin Yvon Raman Spectrometer (Model: LabRAM) with a 632.8 nm He/Ne laser (17 mW), 1800 l/mm grating and an Olympus BX-41 microscope system were used. The spectral collection was performed in the backscattered mode with: ×100 microscope objective, 500 μm pinhole, 500 μm slit width, and a laser power attenuation filter of 10× (i.e., providing a laser power of 1.7 mW). The spectra were collected for a sampling time of 10 s with 10 repeats. All Raman spectra were processed in MATLAB. Background correction was performed through polynomial subtraction, and noise was reduced with a Savitsky–Golay filter. Figure S1 in the Supplementary Material illustrates the effects of the signal processing on raw spectra. The background spectra of the dendrites and the poly-L-lysine (PLL)-modified silicon surface with dendrites are shown in Figure S4. Figure S5 demonstrates the effects of repeated acquisitions on the signal intensity. 

## 3. Results

**Figure 1:**
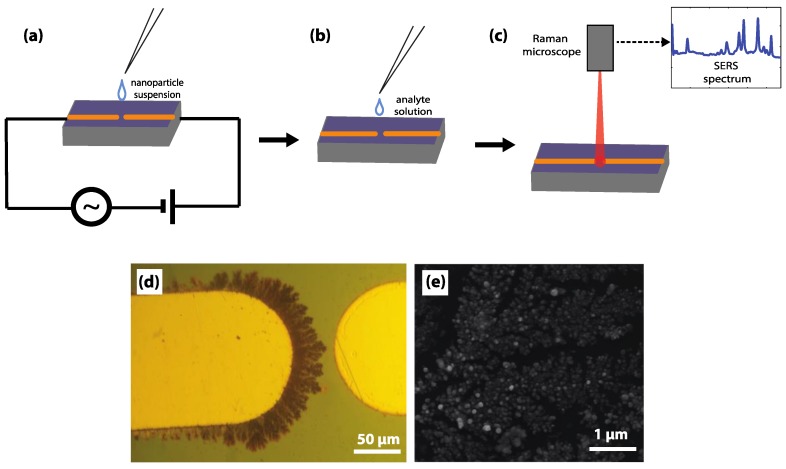
Preparation of the surface-enhanced Raman scattering (SERS) substrate used for detection of food contaminants in liquid food. (**a**) With the nanoparticle solution sitting at the tips of the microelectrodes, the gold microelectrodes are connected to the mixed AC/DC voltage supply to form the SERS-active nanostructures. (**b**) The nanoparticle suspension is removed, and the analyte solution is dropcast upon the surface of the microelectrodes. (**c**) The Raman microscope is used to analyze the gap region between the two microelectrodes, where both the SERS active structures and the analyte have been deposited. (**d**) Between the microelectrode tips, dendritic silver nanostructures grow from the positively biased tip. (**e**) A scanning electron microscopy (SEM) image of the dendritic silver nanostructures.

Figure 1 shows the assembly-to-analysis method applied for detection of trace hazardous materials in common liquid food products. First, the microelectrode chip is connected to a power source at electric field conditions of 10 Hz, 2.9 V peak-to-peak, with an imposed DC bias of 0.5 V (Figure 1a). These conditions have been optimized to create a highly active and sufficiently extensive SERS sensing surface with 12 min of application [13]. Next, a small amount (5 μL) of the prepared analyte solution is placed upon the sensing surface, and allowed to evaporate (Figure 1b). Finally, the solid substrate is placed under the Raman laser, and the SERS spectrum is obtained (Figure 1c). 

Upon application of the nanoparticle suspension, the AC electric field establishes a deterministic dielectrophoretic force on the nanoparticles, directing them to the microelectrode tips. At this low frequency and low voltage condition, dielectrophoresis dominates over electrohydrodynamic flows in the system [13]. Electrohydrodynamic flow (including electro-osmosis and electrothermal flows) can cause bulk fluid flow and mixing effects [14]. Operating in a frequency range that minimizes these effects enables the mass-transfer limited assembly process of nanoparticles into branched, dendritic nanostructures. These “nanodendrites”, shown by the optical microscopy and scanning electron microscopy (SEM) images in Figure 1e,f respectively, have a high density of SERS hotspots, as well as conductive connections between these hotspots which enable broadening of the plasmon resonances necessary for SERS detection [15]. 

**Figure 2:**
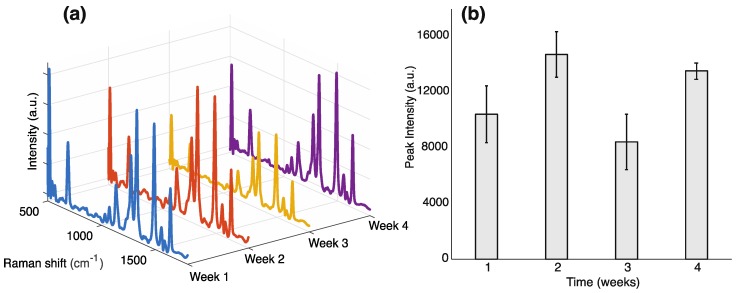
Longevity analysis of the dendritic SERS substrates. Four spectra of R6G were taken every week from simultaneously prepared substrates, for a period of 4 weeks. Week 1 corresponds to time zero (time of dendrite preparation) + 1 week. (**a**) The R6G spectrum was distinctly identifiable every week for 4 weeks. These spectra were taken at a concentration 10^−5^ M, with 10 s acquisition time. (**b**) The average intensity of the key peak at 1360 cm^−1^ was plotted over 4 weeks. The error bars represent the standard deviation of the data.

To characterize the SERS substrates and further understand their potential applicability, we performed a longevity analysis on the substrates. The substrates were prepared simultaneously (on the same day) and consequently tested (one substrate each week) using rhodamine 6G at a concentration of 10^−5^ M as a test analyte. The substrates were stored in room air, in a dark drawer. Over a period of 4 weeks, the substrates maintained their integrity, as shown in Figure 2. The coefficient of variance of the intensity of a key peak at 1360 cm^−1^ (representing in-plane C–H bending [16]) is 25%, indicating that over a significant time period the substrates remain capable of analyte identification, even when stored at atmospheric conditions. We acknowledge that this variance is not insignificant; currently, we are optimizing methods for more homogenous analyte adsorption to reduce this variance and enable more accurate quantification (discussed further in Section 3.2). 

The peak intensity at 1360 cm^−1^ was also used to calculate an enhancement factor, using the equation:

$$EF\;=\;\frac{I_{SERS}/C_{SERS}\;}{I_{NR}/C_{NR}}$$

where *I_SERS_* and *I_NR_* are the peak intensities with SERS and normal Raman respectively (i.e., on a nonmodified silicon oxide microelectrode chip), and *C_SERS_* and *C_NR_* are the concentrations of R6G used in the SERS and normal Raman experiments. Using the 1360 cm^−1^ peak heights in this SERS data and the 1360 cm^−1^ peak height from the normal Raman spectrum shown in Figure S2, the SERS substrates were shown to have enhancement factors ranging from 4.7 × 10^5^–5.9 × 10^5^ across the four-week longevity experiment. 

### 3.1. Thiram Detection in Fruit Juice

**Figure 3:**
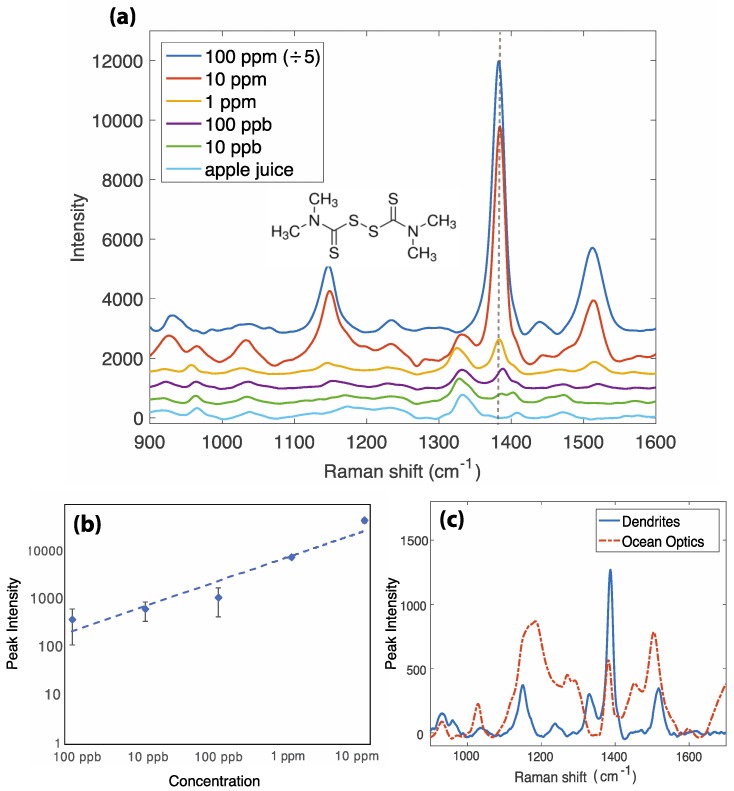
The results for thiram detection in apple juice. (**a**) SERS spectra of thiram at varying concentrations, in unprocessed apple juice. The key peak at 1384 cm^−1^ is used for identification and quantification. (**b**) A log–log calibration curve of thiram in apple juice. Regression analysis yields *y* = 2520⋅*x*^0.5209^, with an R^2^ value of 0.9223. (**c**) A comparison between the SERS spectra of thiram in apple juice on our dendritic SERS surface, and the SERS spectra on Ocean Optics silver SERS substrates.

Thiram is a chemical commonly used as a fungicide and animal repellant in industry and agriculture [17]. Thiram has multiple toxicities—it is a skin and mucous membrane irritant, hepatotoxic, neurotoxic, and teratogenic [18]. The US Environmental Protection Agency dictates 7 ppm (2.9 × 10^−5^ M) as the maximal residue limit for thiram in food products [18]. The currently preferred method of monitoring pesticide concentrations in fruits and vegetables is HPLC [17,19], with reported limits of detection in the low ppb range [17]. Recently, a number of groups have demonstrated the applicability of SERS for the detection of thiram. Zhang et al. showed detection of thiram via SERS on silver nanowires, to a detection limit of 10^−7^ M (24 ppb) [20] and Saute et al. used nanoparticles of differing shapes to achieve a thiram limit of detection (LOD) of 10^−8^ M (2.4 ppb) [21]. A relevant matrix for the real-life detection of thiram is a fruit product, as thiram is frequently used in fruit and soybean farming. Here, we demonstrate a method of separation-free detection of thiram in apple juice. The SERS spectra are shown in Figure 3a, alongside the calibration curve in Figure 3b. The peak at 1384 cm^−1^ is characteristic of thiram, representing the symmetric CH_3_ deformation mode [22]. The peaks at 1150 cm^−1^ and 1530 cm^−1^ are also assigned to thiram, both representing the CN stretching mode (ν(CN)) and the CH_3_ rocking mode (ρ(CH_3_)) [23]. Other key peaks in the samples appear to be due to the apple juice, principally the peak at approximately 1340 cm^−1^. The calibration curve demonstrates a LOD of 115 ppb, or 4.8 × 10^−7^ M. The LOD is calculated as the concentration that corresponds to 3× the standard deviation on the experimental calibration curve. This limit is well below the recommended safe limit, hence this technique is enabling as a method of rapid, cost-effective screening to quickly identify samples with high levels of thiram that may require further quantitative testing. 

To demonstrate the relevance and importance of this technique, we present a comparison to the sensing achievable with a commercially available SERS chip produced by Ocean Optics (Dunedin, FL, USA; item code RAM-SERS-AG-5), which consists of silver nanoparticles supported on a solid substrate. Figure 3c shows the SERS spectra from thiram at a concentration of 1 ppm in pure acetone, on our SERS substrate, and the Ocean Optics silver substrate. Our SERS substrate presents not only the benefit of point-of-use fabrication and reusability, but it also demonstrates increased sensitivity and signal-to-noise ratio compared to this commercially available option. The Ocean Optics substrate is produced upon a paper-based substrate, which may contaminate the Raman spectrum and has a lower density of SERS-active nanostructures than our directed-assembly substrate. 

### 3.2. Detection of Melamine in Milk Products

Melamine is a nitrogenous industrial chemical used in resin production and fertilizer that, upon ingestion and metabolization, precipitates to form nephrotoxic crystals in the kidney, which may lead to acute renal failure [24]. Chronic exposure may be implicated in bladder cancer and infertility [25]. Because of its high nitrogen content (66.7% by mass), melamine may be added illegally to dairy products, infant formulations, or pet food, to boost the apparent protein content [26]. In 2008, this caused the hospitalization of approximately 50,000 infants in China, and in 2007, this caused several hundred deaths of domestic pets in the United States [24,27]. The World Health Organization dictates 2.5 ppm (2.0 × 10^−5^ M) as the maximum residue limit for melamine in milk, and 1 ppm (7.9 × 10^−6^ M) as the maximum residue limit in infant formula [28,29].

The task of detecting melamine in food products is complicated by the fact that it most frequently exists in complex solutions or emulsions, which are often opaque, limiting optical detection methods. Additionally, food products contain several components (e.g., proteins, lipids, and sugars) at much higher concentrations than melamine, which can mask a detection signal and may foul a sensing system. Therefore, the detection of melamine in food solutions typically involves two essential processes: (1) separation/extraction, and (2) detection. 

Presently, the most common academic and industrial methods for the detection of melamine in food products include HPLC with ultraviolet (UV) detection [30,31,32], LC-MS [33,34], infrared (IR) spectroscopy [35], nuclear magnetic resonance (NMR), enzyme immunoassays (EIA) [33,36], capillary electrophoresis [37,38], and recently, Raman spectroscopy and SERS [39,40,41]. Sun et al. provide a recent review of these techniques and their associated limits of detection [3]. The US-FDA recommended analytical methods for melamine detection include HPLC with UV/Vis detection, and LC-MS, as these methods can reach limits of detection as low as 50–250 ppb in liquid milk and infant formula [25,42], well below the recommended limit. However, these methods are often time-consuming, laborious, and require expensive, immovable equipment. 

**Figure 4:**
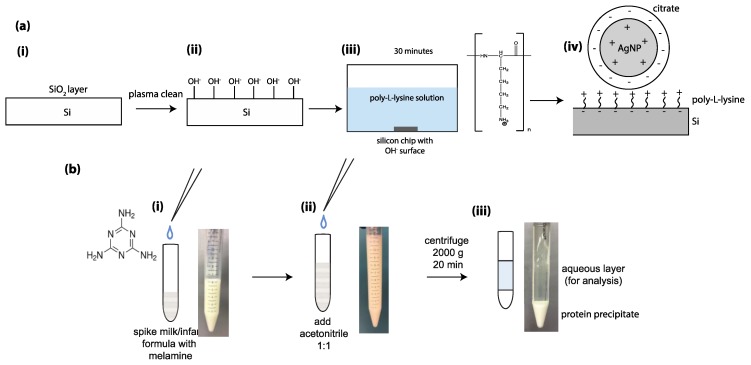
Preparation of the sensing chip and the analyte solutions for detection in milk and infant formula. (**a**) The procedure for silicon surface modification with poly-L-lysine, to anchor the dendrites. (i) The starting silicon surface with a thermally grown oxide layer is plasma cleaned for 3 min, (ii) leaving negatively charged hydroxyl groups at the surface. (iii) The chip is then soaked in poly-L-lysine. (iv) The nanoparticles, coated with a layer of citrate for stabilization in solution, adhere to the poly-L-lysine coated surface. (**b**) The sample processing method applied for milk and infant formula; (i) Spiked solutions of milk/infant formula were thoroughly mixed with a vortex mixer. (ii) Acetonitrile was added to the spiked milk, followed by vortex mixing and centrifugation. (iii) The supernatant (aqueous layer) was removed for analysis, and the protein pellet was discarded.

Here, we present the direct application of our SERS substrates for the detection of melamine in liquid food samples. As our protein precipitation method was not comprehensive, we found that the remaining protein acted as a surfactant to destabilize and dissolve the SERS-active nanostructure. Therefore, as shown in Figure 4a, prior to dendrite growth, we coated the silicon dioxide surfaces with an adsorbed layer of poly-L-lysine (cationic), to electrostatically anchor the citrate-coated nanoparticles (anionic) to the surface. Importantly, the poly-L-lysine monolayer did not present any significant peaks in the range of interest for melamine detection, (see Figure S4). As shown in Figure 4b, the milk and infant formula sample processing was performed by adding acetonitrile in a 1:1 ratio to the melamine-spiked milk solutions. The addition of an organic solvent displaces water molecules surrounding the proteins in milk and lowers the dielectric constant of the medium, promoting aggregation via electrostatic interactions between proteins [43]. Next, the solution was centrifuged, causing the protein components to form a pellet on the bottom of the test tube. The supernatant represents a solution of the aqueous components of milk with acetonitrile and melamine; this was extracted and used for SERS analysis.

**Figure 5:**
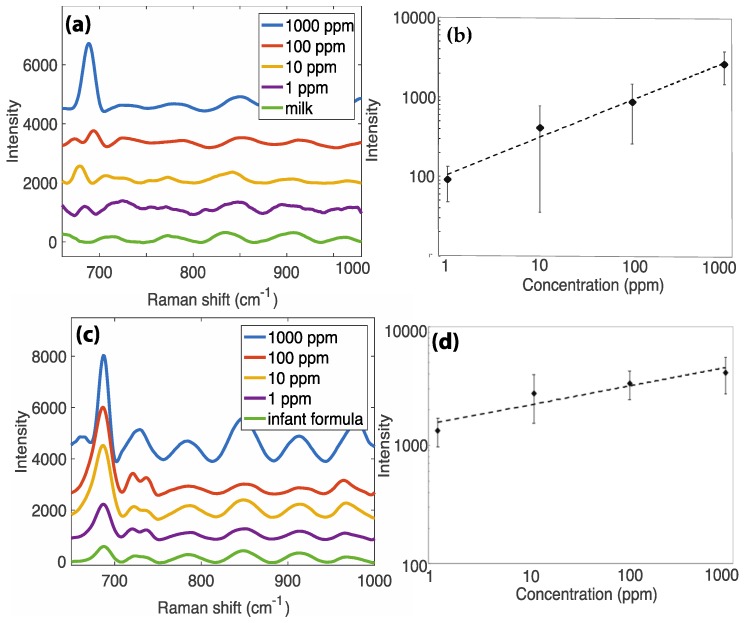
SERS detection of melamine in milk and infant formula. (**a**) The SERS spectra of melamine in milk. The key peak at 686–703 cm^−1^ was used for identification and quantification. (**b**) Log–log plot for calibration of melamine concentration in milk. Points represent an average of n = 5 spectra. Linear regression yields *y* = 106.41⋅*x*^0.4656^, with an R² value of 0.9828. (**c**) The SERS spectra of melamine in infant formula. The key peak at 686–703 cm^−1^ was used for identification and quantification. (**d**) Log–log plot for calibration of melamine concentration in infant formula. Linear regression yields *y* = 1555.8⋅*x*^0.1544^, with an R^2^ value of 0.8883.

The results for melamine in milk and infant formula are shown in Figure 5. Figure S1 in the Supplementary Material shows spectra taken in different locations across the substrate surface, as well as unprocessed SERS spectra, to demonstrate the effects of signal processing in MATLAB. The key peak at 686–703 cm^−1^ is used for calibration, which corresponds to the triazine ring breathing mode of vibration [44]. Notably, the melamine peak appears to shift in different measurements (particularly in the 100 ppm sample of Figure 5a). This may be explained by changes in the solution pH—it has been demonstrated that pH influences the triazine ring breathing mode, shifting its SERS peak from 686 cm^−1^ at low pH (i.e., below the pKa of melamine = 5, when the melamine is protonated) to 703 cm^−1^ at high pH [44]. We also demonstrate this shift in our system through measurements of the melamine peak at differing pH values—the data are shown in Figure S3. The shift observed in the milk analysis may be a result of pH changes in the pre-purchased milk with aging, or from the pre-processing step with acetonitrile. From the calibration curve in Figure 5b for melamine in milk, the theoretical limit of detection is 1.5 ppm (1.2 × 10^−5^ M), below the recommended limit of 2.5 ppm (2.0 × 10^−5^ M). The data does show significant variability, limiting the accuracy in quantitative detection. There are a number of potential sources of this variability, including, potentially differing extraction amounts in the separation process, an inability to completely/effectively extract the protein and lipid components, the inhomogeneity of the SERS surface, and differing distribution of the dropcast analyte across the SERS surface. Rather than a method for rigorous quantitative analysis, we present these results to demonstrate a versatile method for screening-type detection in complex media. We are currently working on methods to improve the sensitivity of the technique via improved separation efficiency and directed analyte deposition. Increasing the of spectra analyzed should also decrease the variability and enable more effective quantification. 

Melamine detection in infant formula is perhaps even more societally relevant, as infant formula is a synthetic food product with a significant history of hazardous contamination [28,45]. We applied an identical protein precipitation and surface modification method with melamine in infant formula (as shown in Figure 4b); that is, we precipitated the proteins with acetonitrile and coated the silicon surface with poly-L-lysine to prevent the dendrites from dissolving. Notably, this process appeared more effective in infant formula. As shown in Figure 5d, the melamine peaks are stronger, and visually, the supernatant appeared less cloudy. In this way, we successfully detected melamine in infant formula across four orders of magnitude, achieving a theoretical limit of detection of 105 ppb (8.3 × 10^−7^ M). This figure is well below the WHO recommended limit in infant formula of 1 ppm (7.9 × 10^−6^ M) [42]. 

## 4. Conclusions

We have demonstrated a method for the detection of toxic contaminants in complex food matrices, with limited sample processing requirements, using a versatile SERS platform produced through electrokinetic deposition of silver nanoparticles. The presented technique achieves detection of thiram in apple juice and melamine in milk and infant formula at levels below limits recommended by the US-EPA and WHO respectively, and is therefore sufficient for screening applications to quickly identify samples that exceed these limits. We anticipate that these limits of detection may be further improved by methods including surface modification of nanoparticles with selective molecules [46], coatings to promote hydrophobic analyte concentration upon the detection site [47], chemometrics for better spectral analysis [48], and coupled microextraction methods for better analyte enrichment. 

## Supplementary Materials

The following are available online at http://www.mdpi.com/1424-8220/18/8/2726/s1.
