# Supplementary material for: Direct Detection of Toxic Contaminants in Minimally Processed Food Products Using Dendritic Surface-Enhanced Raman Scattering Substrates
Source: Sensors (Basel). 2018 Aug 19;18(8):2726. doi: 10.3390/s18082726 (PMC6111781; doi:10.3390/s18082726)
Supplement: Supplementary file 1 [file sensors-18-02726-s001.pdf]

Supplementary Materials:

Direct Detection of Toxic Contaminants in Minimally Processed Food Products Using Dendritic Surface-enhanced Raman Scattering Substrates

Hannah Dies [1], Maria Siampani [2], Carlos Escobedo [1], and Aristides Docoslis [1,*]

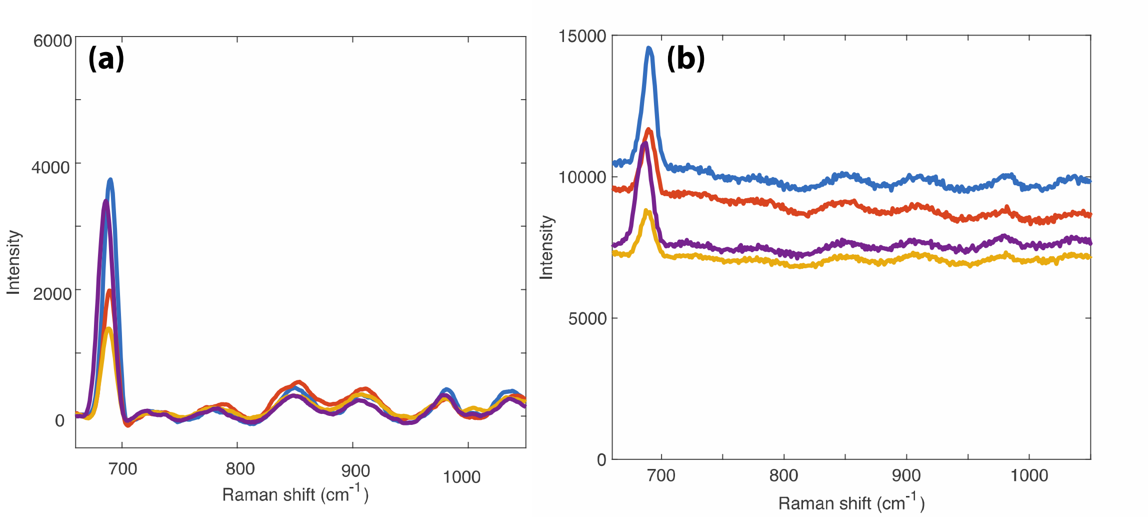

Figure S1. Spectra for 1000 ppm melamine in milk. (**a**) The individual spectra with signal processing performed (polynomial background subtraction + Savitsky-Golay filter), all at 1000 ppm, on different locations across the dendritic surface of the SERS substrate. (**b**) The pre-processed (raw) spectra from (**a**).

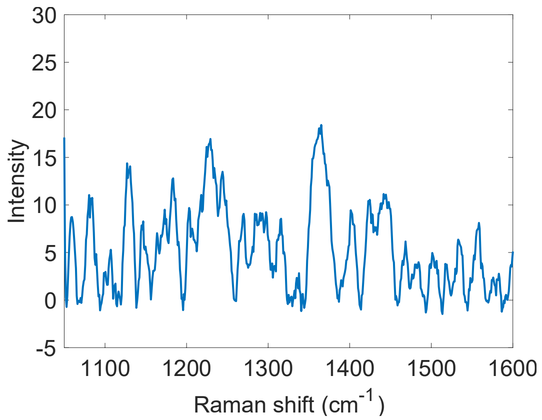

Figure S2. The normal Raman spectrum of R6G at 10 mM on a silicon chip. The dominant peak at 1360 cm^{-1} was used for calculation of the enhancement factor.

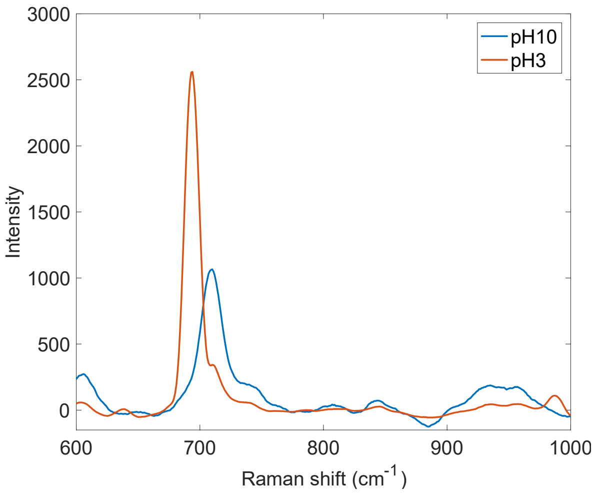

Figure S3. Shifting of the melamine peak with pH. Both spectra are taken at 100 ppm in water, with HCl and NaOH used to adjust the pH of the sample. The melamine peak shifts from 686 cm^{-1} to 703 cm^{-1} with an increase in pH from 3–10 (the pK_a of melamine is 5.0).

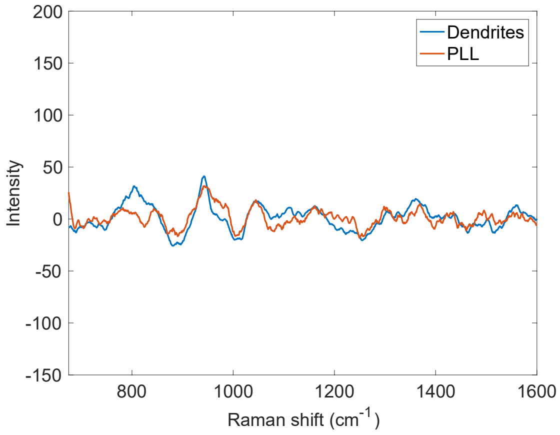

Figure S4. The SERS background of the sensing surface, with dendrites (silver nanostructures with a citrate capping, shown in blue), and a pre-assembled PLL monolayer beneath the dendrites (shown in red). The spectra were taken with 10s acquisition time.

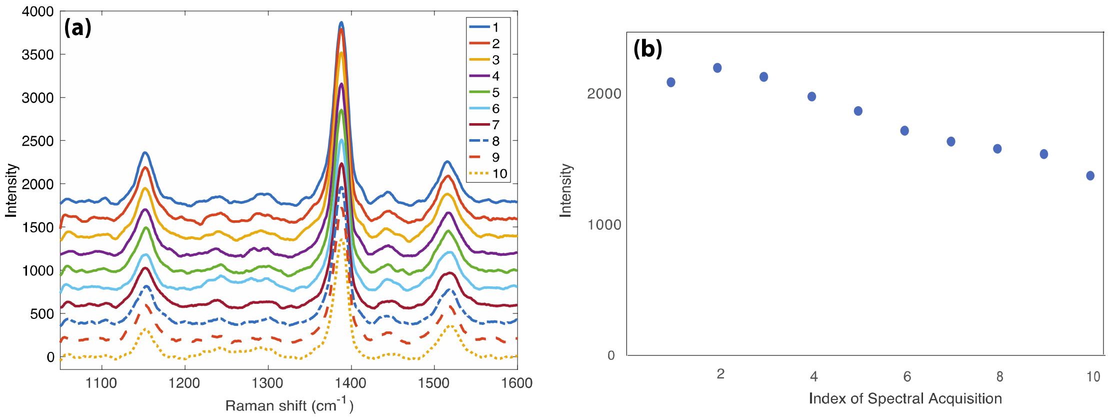

Figure S5. Demonstration of signal changes with repeated spectral acquisitions, for thiram detection in acetone. (**a**) Spectra of thiram at 10 ppm in acetone, with 10 s of acquisition time each. The spectra are indexed 1–10, indicating the sequence of acquisition. (**b**) Notably, there is some signal decrease (measured by the intensity of the 1384 cm^{-1}) peak, as the acquisition continues, likely attributed to photobleaching of the analyte.
